# Editorial Notes

**Published:** 1905-06

**Authors:** 


### Editorial Notes.

**Sanatorium Treatment of Phthisis.**

THIS subject has been much discussed of late, but there are indications that it is not yet sufficiently understood either by the profession or the philanthropic public: it is even less realised by the patient what are its uses and limits. We read much of the utility, we read also of the futility, of sanatoria; optimists claim too much, and pessimists decline to admit that the results are worth the labour and expense. A consideration of the disease from the pathological standpoint must show at once that only a limited number can do well under any treatment: the public and the patient, with no pathological knowledge, are apt to lead astray even the physician, who should be able to exercise a pathological scrutiny of the patient and form his judgment accordingly. It has now been abundantly proved that a natural immunity can be encouraged by perfect hygienic conditions, or discouraged by the reverse of these, and that even when that immunity has been lost it may be again regained under better surroundings. Cases can be cured, and the best treatment is that afforded by an open-air sanatorium.

There can be no doubt that sanatorium treatment is getting much discredit by the inefficiency with which it is often attempted, and by the want of selection of suitable cases. It is often repeated that open-air treatment can be carried out at home: perhaps so, after a patient has already learnt at a sanatorium what and how it is to be done, and this is one of the most important uses of the sanatorium—to teach the patients how to conduct themselves at home; but they must first have a start at a sanatorium, and even then the inadequacy of the subsequent home treatment is doing much to discredit the work done at sanatoria for the poorer classes. As regards the better class of patients, there is less need for careful selection of the cases: the reputation of the method is nothing to the patient who is seeking cure, and length of residence is of no consequence to those who can afford to pay for that kind of life. It is the usual experience that advanced cases are expensive and benefit little, whereas early cases cost less and greatly improve.

But as regards the poorer classes, it is necessary that the cases should be in the early stages if they are to derive any benefit from the short stay which is usually allowed; for cases of a more advanced kind, it is essential that they should continue treatment until they are practically beyond any danger of relapse. The great needs, then, are two—incipient cases and ample funds for maintenance. It becomes, therefore, needful that if a sanatorium for the poor is to achieve any permanent results there must be some machinery by which there shall be an adequate selection of the cases, otherwise the sanatorium must gradually become a home for the incurables. It is of the highest economic importance, and of national importance, that we should obtain the maximum benefit from our sanatoria, and to do this we have to recognise that the beds must not be occupied by those patients who can obtain no permanent benefit to the exclusion of those who are likely to be permanently cured.

As regards the sanatorium for the poorer classes which has been established at Winsley for the three adjoining counties, it has already been found necessary that the promoters should exercise due care in the selection of cases for admission. One difficulty therein arises from the fact that patients are admitted on the reports of two medical men, who may not yet realise the necessity for a very careful selection. The certificate of the medical attendant is the first thing needful, and then a second report is required from some medical man who examines for the Medical Board, and who is supposed to know what the views of the Board may be. Armed with these two certificates of fitness, the patient's name is placed on the list for an early vacancy. The following code of instructions is forwarded to the medical attendant, and it is hoped that the medical examiners of candidates will use every effort to assist the Board of Management in their endeavours to make the sanatorium a brilliant success:—

You are requested to read carefully the "Classification of Cases" adopted, and the suggestions as regards "Suitability of Cases."

The following is the "Classification of Cases" adopted:—

I. "Good Cases." Full working capacity may be restored without much fear of relapse if reasonable care is taken.

II. "Hopeful Cases." A good chance of regaining working capacity exists, although not perhaps in four months' treatment. Risk of relapse greater, caution will be needful for a longer time.

III. "Some benefit may be expected." Cases of long standing, and with much lung affected. Improvement more or less lasting according to the life led. Will only be fit for very easy, light work under favourable conditions.

IV. "Unsuitable Cases."

The following suggestions as regards "Suitability of Cases" should be borne in mind:—

I. The most suitable case for a course of treatment limited to sixteen weeks is one newly detected, and found to have nothing more than signs of consolidation at one apex, or some similar lesion.

II. Cases with a greater extent of one lobe of one lung affected. These may do sufficiently well.

III. Cases with fair extent of disease in one lobe and slight disease in a second might be admitted, but only if otherwise the indications are favourable, and if there is a prospect of carrying out after-treatment for the requisite time.

IV. Cases complicated with laryngeal disease should be admitted only if the affection of both larynx and lung is in an early stage.[1]

V. Cases with disease dating back for several years should be excluded.

[1] N.B. A detailed description of the site and extent of the laryngeal disease is desirable.

Your report in the case should be sent under closed cover to any member of the Medical Consultative Board whom you may select, and to whom the patient is to go for the second certificate as to fitness for sanatorium treatment at Winsley.

**Tuberculin Redivivus.**

There are indications of a revival of the much-abused but also greatly lauded tuberculin. A paper by Dr. Batty Shaw[1] gives the reasons why the earlier tuberculin introduced with such a flourish of trumpets in 1890 was discarded. A similar fate pursued the second effort in 1897. With the exception of a few investigators like Dr. Heron, who in 1901 gave his results of the continued use both of tuberculin and tuberculin R, few physicians continued to use either one or the other, excepting very occasionally for purposes of diagnosis in obscure cases of possible tuberculosis.

Since the British Congress on Tuberculosis in 1901 there seems to have arisen a more general desire to review the question anew; it has been thought that the original tuberculin was unduly blamed, and that the newer varieties, whilst free from the harmfulness of the former, still retained some of its usefulness. Accordingly reports and results of extended and long-continued inquiries are now accumulating, and a revival of interest in the subject is to be encouraged.

Dr. Shaw endeavours to show "what are the proper indications for tuberculin treatment, and also that with proper management the case cures may be considerably increased in number by the adoption of the tuberculin treatment, combined with the other general methods already so well carried out in the sanatoria of this country," and elsewhere.

It is now becoming the orthodox belief that the cases suitable for tuberculin are the earliest cases, and those in which the disease is limited to one apex; the afebrile, the well-nourished, and those in which the disease shows no indication of active progress—these are the cases which are said to do well with tuberculin; they are also the cases which do well

[1] *Clin. J.*, 1905, xxvi. 38.

under sanatorium life without tuberculin: hence it appears that tuberculin is to be in active competition with non-medicinal hygienic measures, and the more advanced cases are still left outside the pale. Formerly it was thought that even advanced cases would do well under the influence of tuberculin, but now we are told that these cases are not suitable ones for tuberculin, neither are they for the sanatorium. The early cases may do well by either method: it is for the sanatorium authorities to decide the question whether hygienic measures alone will suffice, and whether the tuberculin should be reserved for those cases which do not manifest any or sufficient improvement after trial of sanatorium hygienic routine.

